# Epithelial immunomodulation by aerosolized Toll-like receptor agonists prevents allergic inflammation in airway mucosa in mice

**DOI:** 10.3389/fphar.2022.833380

**Published:** 2022-08-29

**Authors:** David L. Goldblatt, Gabriella Valverde Ha, Shradha Wali, Vikram V. Kulkarni, Michael K. Longmire, Ana M. Jaramillo, Rosha P. Chittuluru, Adrienne Fouts, Margarita Martinez-Moczygemba, Jonathan T. Lei, David P. Huston, Michael J. Tuvim, Burton F. Dickey, Scott E. Evans

**Affiliations:** ^1^ Department of Pulmonary Medicine, University of Texas MD Anderson Cancer Center, Houston, TX, United States; ^2^ Howard Hughes Medical Institute, Chevy Chase, MD, United States; ^3^ University of Texas Rio Grande Valley School of Medicine, Edinburg, TX, United States; ^4^ Department of Microbial and Molecular Pathogenesis, Texas A&M Health Science Center, Houston, TX, United States; ^5^ Clinical Science and Translational Research Institute, Texas A&M Health Science Center, Houston, TX, United States

**Keywords:** toll-like receptors, allergic asthma, allergen immunotherapy, innate immunity, lung epithelial cells, allergen tolerance

## Abstract

Allergic asthma is a chronic inflammatory respiratory disease associated with eosinophilic infiltration, increased mucus production, airway hyperresponsiveness, and airway remodeling. Epidemiologic data reveal that the prevalence of allergic sensitization and associated diseases has increased in the twentieth century. This has been hypothesized to be partly due to reduced contact with microbial organisms (the hygiene hypothesis) in industrialized society. Airway epithelial cells, once considered a static physical barrier between the body and the external world, are now widely recognized as immunologically active cells that can initiate, maintain, and restrain inflammatory responses, such as those that mediate allergic disease. Airway epithelial cells can sense allergens *via* expression of myriad Toll-like receptors (TLRs) and other pattern-recognition receptors. We sought to determine whether the innate immune response stimulated by a combination of Pam2CSK4 (“Pam2”, TLR2/6 ligand) and a class C oligodeoxynucleotide ODN362 (“ODN”, TLR9 ligand), when delivered together by aerosol (“Pam2ODN”), can modulate the allergic immune response to allergens. Treatment with Pam2ODN 7 days before sensitization to House Dust Mite (HDM) extract resulted in a strong reduction in eosinophilic and lymphocytic inflammation. This Pam2ODN immunomodulatory effect was also seen using Ovalbumin (OVA) and A. oryzae (Ao) mouse models. The immunomodulatory effect was observed as much as 30 days before sensitization to HDM, but ineffective just 2 days after sensitization, suggesting that Pam2ODN immunomodulation lowers the allergic responsiveness of the lung, and reduces the likelihood of inappropriate sensitization to aeroallergens. Furthermore, Pam2 and ODN cooperated synergistically suggesting that this treatment is superior to any single agonist in the setting of allergen immunotherapy.

## Introduction

Epidemiologic data demonstrate that the prevalence of allergic sensitization and associated diseases has increased in the 20th century. This has been hypothesized to be partly due to reduced contact with microbial organisms (the hygiene hypothesis) in industrialized society (Lambrecht and Hammad, 2017). In one study comparing Amish and Hutterite children, higher levels of microbial elements in farm dust were found to be strongly protective against developing asthma (Stein et al., 2016). In mechanistic studies, this effect was found to be mediated by downregulation of inflammatory pathways within airway epithelial cells (Schuijs et al., 2015).

Allergic inflammation is the principle driving force behind diseases such as asthma, rhinitis, and dermatitis (Hammad and Lambrecht, 2021). Across these allergic diseases, eosinophilic infiltration in tissues and epithelial responses to IL-13 are consistently observed, frequently in manifestations that are highly dependent on tissue-specific cellular differentiation.

Airway epithelial cells, once considered static physical barriers between the body and the external world, are now widely recognized as immunologically active cells that can initiate, maintain, and restrain inflammatory responses, such as those that mediate allergic disease (Hammad and Lambrecht, 2021). Lung airway epithelial cells express myriad pattern recognition receptors (PRRs) such as Toll-like receptors (TLRs) that enable them to sense and respond to a variety of external triggers, usually referred to as pathogen-associated molecular patterns (PAMPs). The epithelial-derived cytokines IL-33, thymic stromal lymphopoietin (TSLP), and IL-25 are widely recognized as alarmins produced in response to allergens, such as those associated with house-dust mites (HDM). These cytokines activate leukocytes involved in allergic inflammation, such as eosinophils, T helper 2 (T_H_2) cells, mast cells, basophils, type 2 innate lymphoid cells (ILC2), and dendritic cells (DC), which work together to polarize the immune response in a type 2 direction.

Our group has studied means of stimulating lung epithelial cells to defend against microbial pathogens (Evans et al., 2011). We have shown that a combination of Pam2CSK4 (“Pam2”, a TLR2/6 ligand) and a class C oligodeoxynucleotide ODN362 (“ODN”, a TLR9 ligand), when delivered together by aerosol (“Pam2ODN”) synergistically activate an innate immune response in the lung mucosa (but not systemically) which results in protective host resistance to bacteria, fungi, and viruses (Clement et al., 2008; Tuvim et al., 2009a; Evans et al., 2010; Duggan et al., 2011). Pam2ODN-mediated pathogen resistance is initiated very rapidly and has been shown to be mediated by lung epithelial cells (Cleaver et al., 2014).

More recently, we showed that Pam2ODN can attenuate chronic asthma-like lung disease in mice infected with Sendai virus (SeV) (Goldblatt et al., 2020). As hypothesized, Pam2ODN exerted a strong protective effect against the chronic disease by reducing the viral burden in the acute infection. However, we also observed efficacy when Pam2ODN treatment was delivered outside the time range when reduction in viral burden could account for the anti-allergic effect. This apparent dissociation between the reduction in viral burden and chronic disease severity led us to hypothesize that Pam2ODN also modulated the type 2 immune response to the viral infection that drives the chronic inflammatory disease.

To disentangle antimicrobial and immunomodulatory effects of Pam2ODN, we present data here from allergic models where pathogen killing is irrelevant. In this study, we show that Pam2ODN attenuates the type 2 allergic immune response to HDM, Ovalbumin (OVA), and *Aspergillus oryzae* (Ao) in the lung mucosa.

## Results

### Pam2ODN prevents allergic inflammation to HDM

**FIGURE 1:**
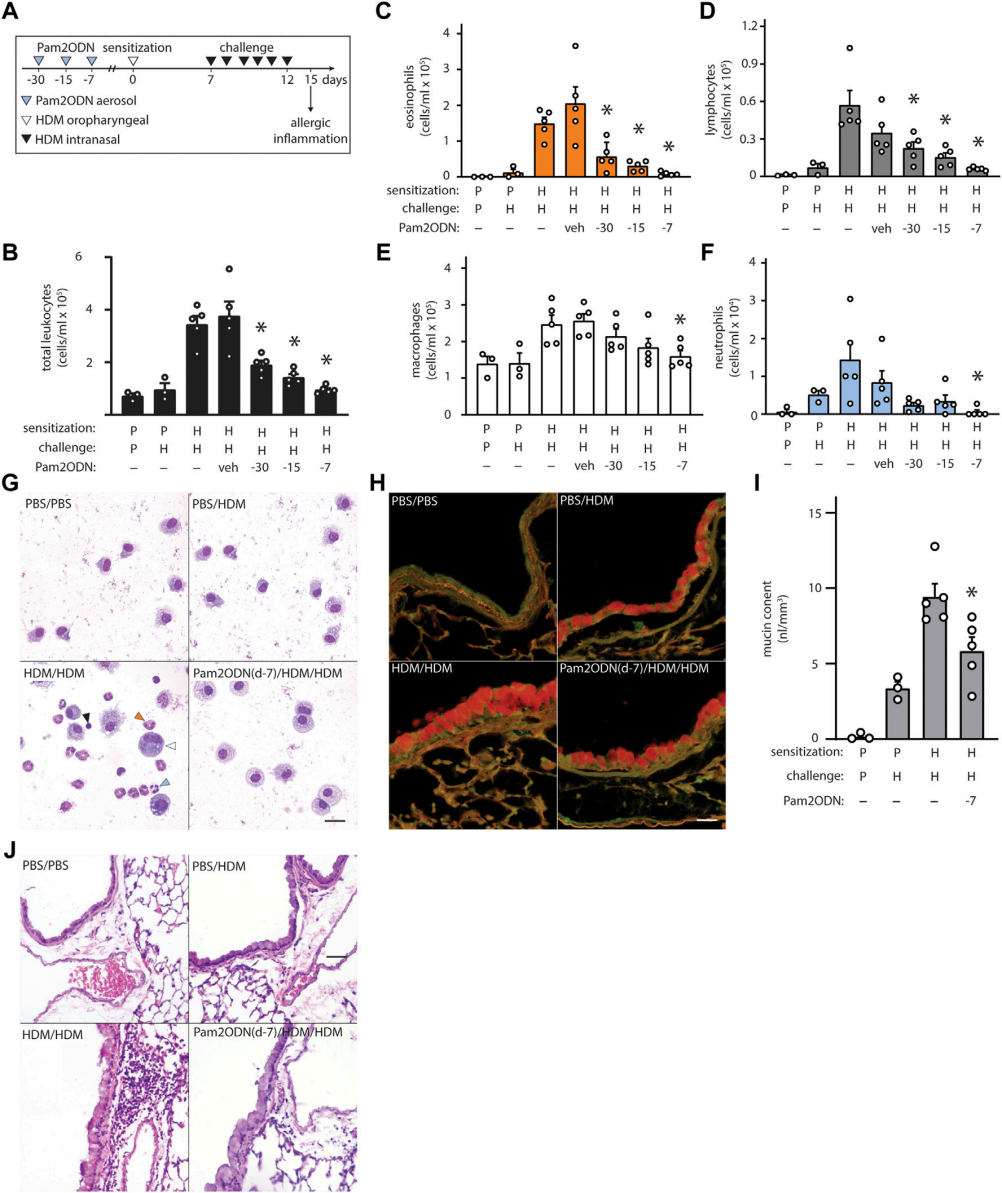
Exposure to Pam2-ODN attenuates allergic inflammation to HDM. **(A)** HDM experimental paradigm with a sensitization of 100 µg HDM and challenges of 10 µg HDM. In these studies, a single treatment of Pam2ODN was delivered by aerosol as described, 30-, 15-, and 7-days before initial HDM sensitization to naïve mice. **(B)** Leukocytes obtained by lung lavage were pelleted onto glass slides by centrifugation and stained with Wright-Giemsa. Scale bar = 20 μm. **(C–F)** Quantification of leukocytes in lung lavage for **(C)** eosinophils, **(D)** lymphocytes, **(E)** macrophages, and **(F)** neutrophils (N = 3-5 mice). **(G–H)** Airways stained with H&E to demonstrate submucosal inflammation. Scale bar = 40 μm **(I)** Airway epithelium stained with PAFS to demonstrate intracellular mucin in red. Scale bar = 20 μm. **(J)** Quantification of intracellular mucin content by image analysis of airway (N = 3-5 mice). Bars show mean +/- SEM. P* < 0.05 by one-way ANOVA with Dunnett’s test for multiple comparisons against [HDM/HDM] control. *p* = PBS. H = HDM.

Mice were sensitized by airway instillation of 100 μg HDM extract at day 0, followed by 6 daily challenges of 10 μg HDM extract from day 7–12, and evaluation of allergic inflammation on day 15 (Figure 1A) (Willart et al., 2012). As previously reported, HDM-sensitized, HDM-challenged (HDM/HDM) mice exhibited robust allergic inflammation, though PBS-sensitized, HDM-challenged (PBS/HDM) did not (Supplementary Figure S1). Allergic inflammation to HDM was reflected by the substantial influx of eosinophils (Figure 1G, orange arrowhead) and lymphocytes (Figure 1G, black arrowhead) with small numbers of neutrophils (Figure 1G, blue arrowhead). Macrophages were increased only slightly in number but were observed to be larger and more intensely stained than PBS/PBS, indicating activation (Figure 1G, open arrowhead).

When mice were treated with a single Pam2ODN treatment 7 days before HDM sensitization, we observed large reductions in leukocytes collected in lung lavage fluid, compared to HDM/HDM mice (Figure 1B). Differential cell quantification with Wright-Giemsa stain revealed a >90% decrease in eosinophils (Figure 1C), >85% decrease in lymphocytes (Figure 1D), >30% decrease in macrophages (Figure 1E), and >95% decrease in neutrophils (Figure 1F). Pam2ODN treatments similarly showed efficacy when administered 30- and 15-days before sensitization, with a trend toward less efficacy when the time between treatment and sensitization was increased. This trend is best observed when leukocyte quantification of multiple experiments is combined (Supplementary Figures S2A–E).

On H&E staining of lung tissue, most of the infiltrating leukocytes were localized to the submucosal space between major airways and vasculature, though some alveolar inflammation was also observed (Figure 1J, Supplementary Figure S2F). Mucous metaplasia of lung epithelial cells was assessed by staining lung tissue with fluorescent PAS stain (PAFS) (Figure 1H) (Piccotti et al., 2012). Quantitative image analysis of intracellular mucin content revealed that intracellular mucin accumulates moderately in PBS/HDM mice despite the near-complete absence of inflammatory leukocytes, though HDM sensitization was required for the full phenotype (Figure 1I). A single Pam2ODN treatment 7 days before HDM sensitization reduced intracellular epithelial mucin content >35%.

### Pam2ODN blocks sensitization to HDM in the airway

**FIGURE 2:**
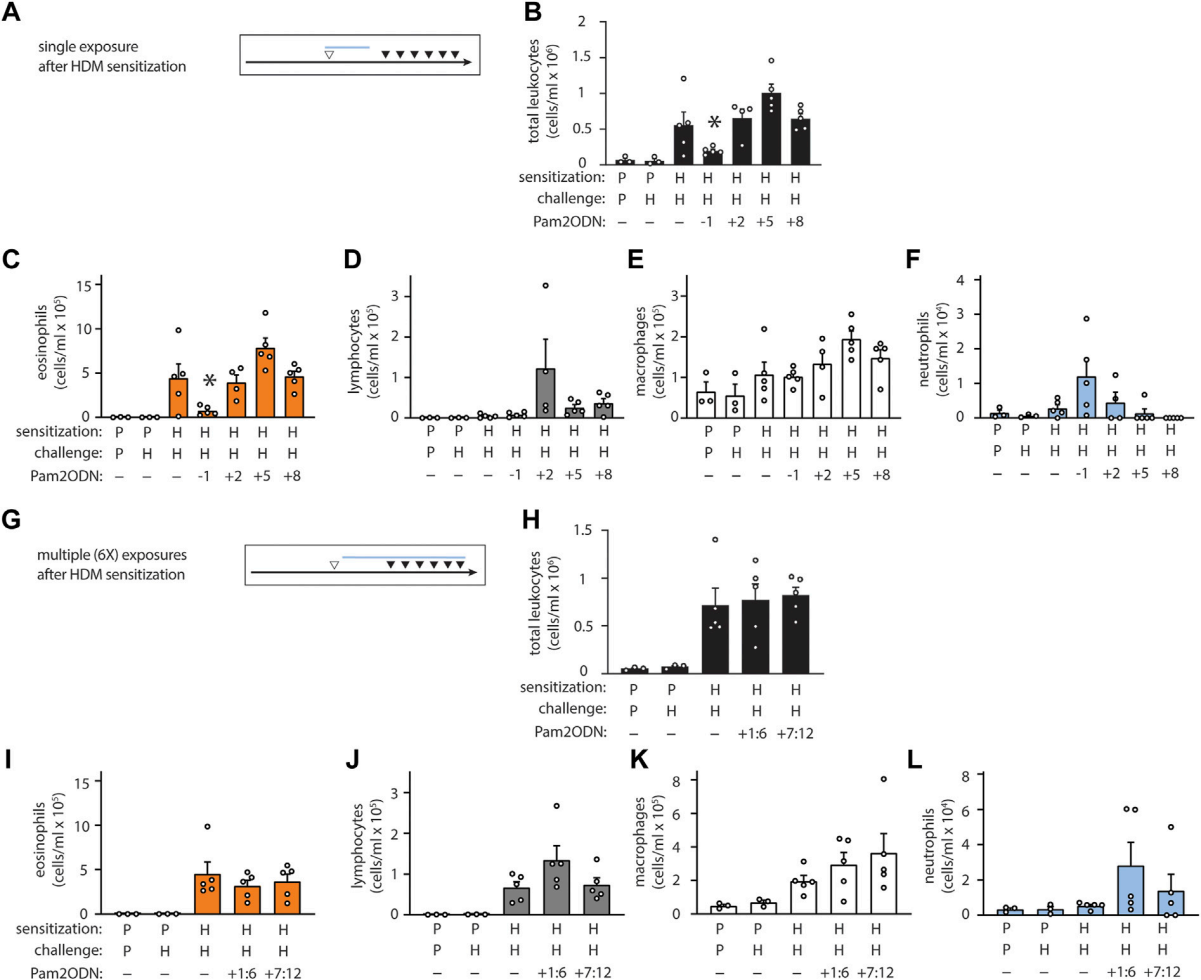
Pam2-ODN prevents sensitization to HDM. **(A,G)** Quantification of leukocytes in lung lavage when a single Pam2-ODN treatment was administered before or after sensitization to HDM (N = 3-5 mice). **(B,H)** Quantification of lung leukocytes when 6 consecutive daily Pam2-ODN treatments were administered after sensitization to HDM during the first week (days +1:6) or concurrently with challenge (days +7:12). Quantification of leukocytes in lung lavage for **(C,I)** eosinophils, **(D,J)** lymphocytes, **(E,K)** macrophages, **(F,L)** neutrophils. Pictograms show blue bar at the approximate range of Pam2-ODN exposure in relationship to sensitization and challenge. Bars show mean +/- SEM. P* < 0.05 by one-way ANOVA with Dunnett’s test for multiple comparisons against [HDM/HDM] control. *p* = PBS. H = HDM.

We next evaluated whether Pam2ODN prevented sensitization to HDM or attenuated allergic inflammation during the challenge phase. To do this, we varied the time-interval and the relative chronological order of Pam2ODN treatment and HDM sensitization. When Pam2ODN treatment was administered just 1 day before HDM sensitization, eosinophils were still significantly reduced, but this effect was completely absent when treatment was administered just 2 days after HDM sensitization, and at any later time point (Figures 2A–F). Since multiple treatments of Pam2ODN had higher efficacy than a single treatment (Supplementary Figure S3), we evaluated whether the lack of Pam2ODN efficacy after HDM sensitization could be due to insufficient treatment by administering 6 daily Pam2ODN treatments after HDM sensitization. As was observed using single treatments, multiple treatments after HDM sensitization did not reduce allergic inflammation (Figures 2G–L).

Historically, most preclinical investigation of allergic disease has been carried out using OVA. This model differs significantly from HDM because sensitization occurs intraperitoneally, followed by airway mucosal challenge. To better establish the breadth of the anti-allergic effects of Pam2ODN, we also evaluated whether Pam2ODN could prevent airway allergic inflammation in the setting of systemic HDM sensitization occurring at a different location than the lungs. To do this, we used the same time schedule for HDM sensitization and challenge as previously shown, except that HDM sensitization was performed by intraperitoneal injection of 100 μg HDM (Supplementary Figure S4A). Pam2ODN treatments were administered at day 0 and day 6 to cover the entire challenge period, however this resulted in no significant reduction in allergic inflammation (Supplementary Figures S4B–E).

### Pam2 and ODN interact synergistically

**FIGURE 3:**
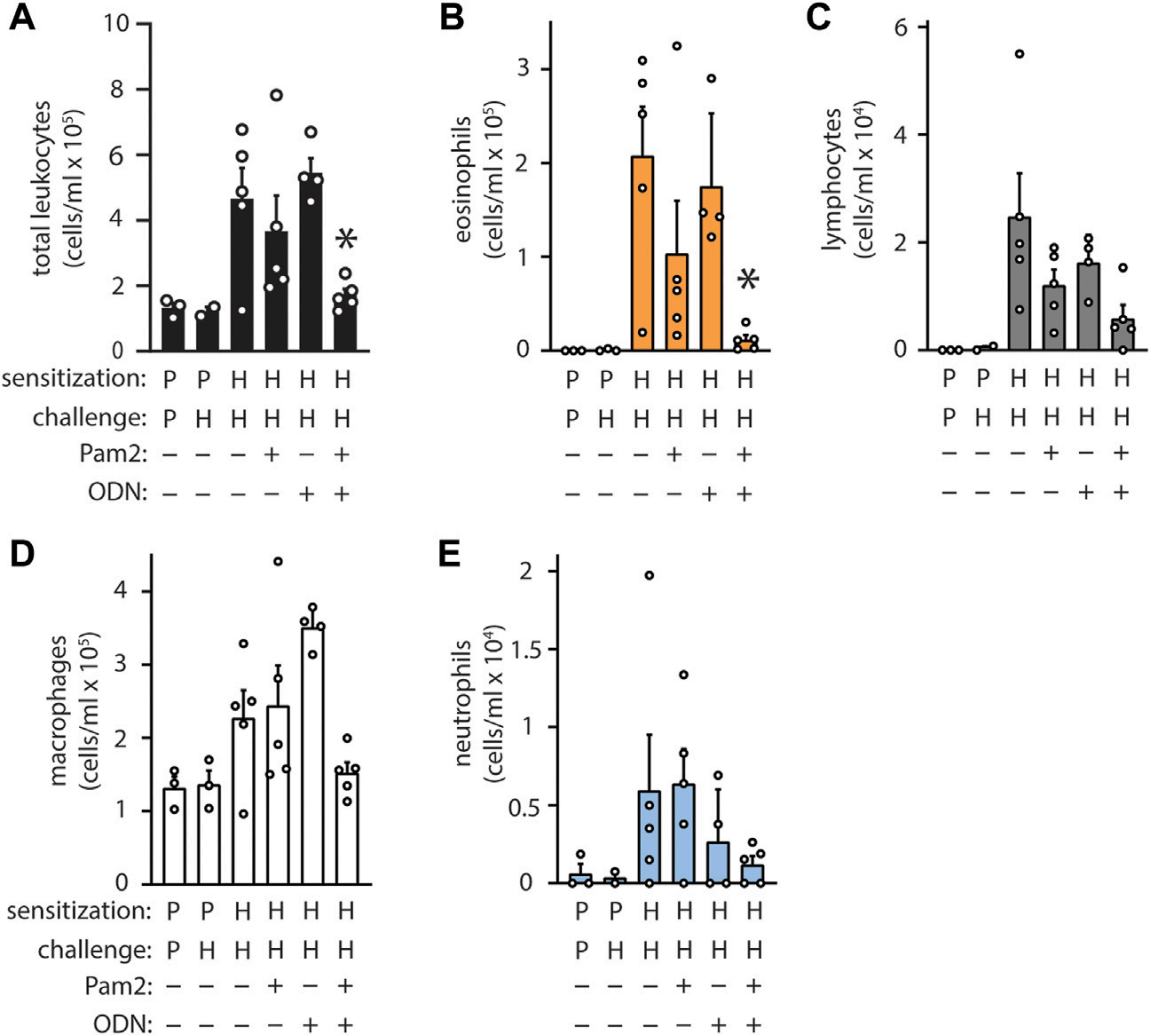
Pam2 and ODN interact synergistically to block sensitization to HDM. Pam2, ODN, or Pam2ODN was administered 7 days before sensitization to HDM. Pam2 and ODN treatments were performed in an identical manner and at identical concentrations as Pam2ODN treatments throughout. Quantification of leukocytes in lung lavage for **(A)** total leukocytes **(B)** eosinophils, **(C)** lymphocytes, **(D)** macrophages, and **(E)** neutrophils (N = 3-5 mice). Bars show mean +/- SEM. P* < 0.005 for synergy between Pam2 and ODN by linear regression. *p* = PBS. H = HDM.

The ligands Pam2 and ODN were selected for their remarkable synergy, and their dosing was established based on maximal pneumonia-protective effect, (Alfaro et al., 2014), however this characteristic has only been evaluated for antimicrobial resistance thus far (Duggan et al., 2011). To evaluate whether Pam2 and ODN also cooperate for allergic immunomodulation, the agonists were aerosolized individually or in combination 7 days before HDM sensitization. Neither Pam2 nor ODN exhibited any significant efficacy when administered alone, but the combination Pam2ODN treatment completely blocked allergic inflammation (Figures 3A–E).

### Pam2ODN attenuates allergic inflammation to OVA

**FIGURE 4:**
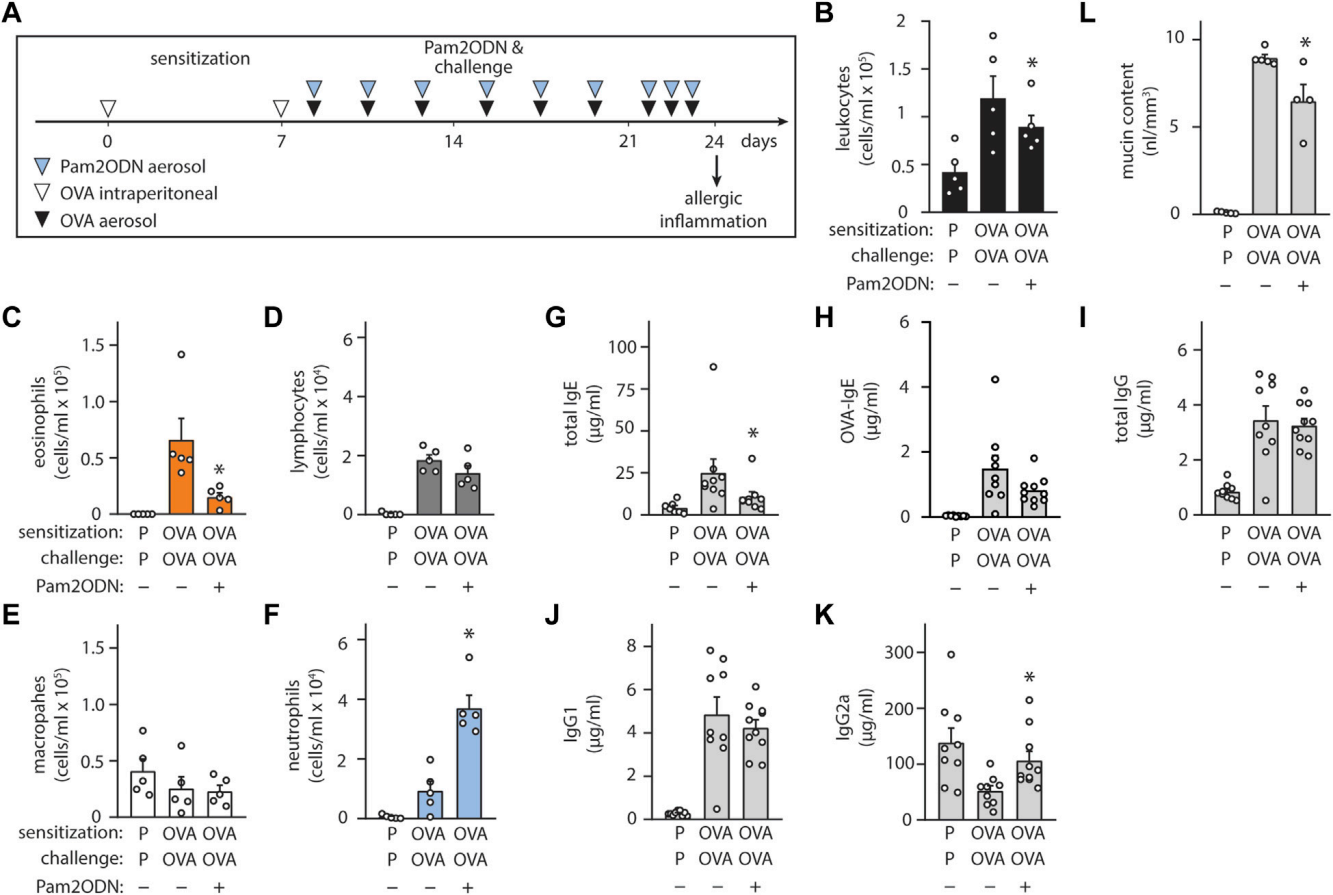
Exposure to Pam2-ODN attenuates allergic inflammation to OVA. **(A)** OVA experimental paradigm with sensitization of 20 µg OVA by intraperitoneal injection and aerosol challenges of 2.5% OVA. **(B–F)** Quantification of leukocytes in lung lavage for **(B)** total leukocytes **(C)** eosinophils, **(D)** lymphocytes, **(E)** macrophages, and **(F)** neutrophils (N = 4-5 mice). **(G–J)** Quantification of serum immunoglobulin concentrations by ELISA for **(G)** IgE, **(H)** OVA-specific IgE, **(I)** IgG, **(J)** IgG1, and **(K)** IgG2a (N = 8-9 mice). **(L)** Quantification of intracellular mucin content by image analysis of airway stained with PAFS (N = 4-5 mice). Bars show mean +/- SEM. p* < 0.05 by unpaired students’ *t* test for OVA vs. OP-OVA. P = PBS. OVA = Ovalbumin.

To evaluate the generalizability of Pam2ODN allergic immunomodulation, we also tested a conventional 3-week OVA model (Figure 4A), consisting of 2 sensitizing intraperitoneal injections during the first week, and aerosol challenges beginning on day 8 administered every 2–3 days until day 21. From day 21 to day 24, OVA aerosol challenges were given daily, and allergic inflammation was assessed at day 24. Pam2ODN treatments were given immediately before OVA challenges. OVA-sensitized, OVA-challenged (OVA/OVA) mice had elevated leukocytes, compared to PBS-sensitized, OVA-challenged (PBS/OVA) mice (Figure 4B). Pam2ODN treatment reduced eosinophils >75% (Figure 4C), but macrophages (Figure 4E) and lymphocytes (Figure 4D) were unchanged. With this Pam2ODN treatment model, neutrophils were observed to be elevated (Figure 4F), however this is likely a direct response to Pam2ODN, which elicits neutrophilic inflammation over a period of 72 h, rather than a secondary effect on the allergic response (Alfaro et al., 2014). Airway epithelial mucin content was also decreased >25% by Pam2ODN treatment (Figure 4L).

Assessing changes in the systemic immune response, Pam2ODN treatment reduced serum immunoglobulin E (IgE) levels by >50% (Figure 4G), including a strong trend towards reducing OVA-specific IgE levels (Figure 4H), but total IgG (Figure 4I) and IgG1 (Figure 4J) were unchanged. IgG2a levels, which increase with type 1 immune responses, decreased in OVA/OVA mice, compared to PBS/PBS, and Pam2ODN treatment reversed this change (Figure 4K).

### Pam2ODN attenuates allergic inflammation to Aspergillus oryzae

**FIGURE 5:**
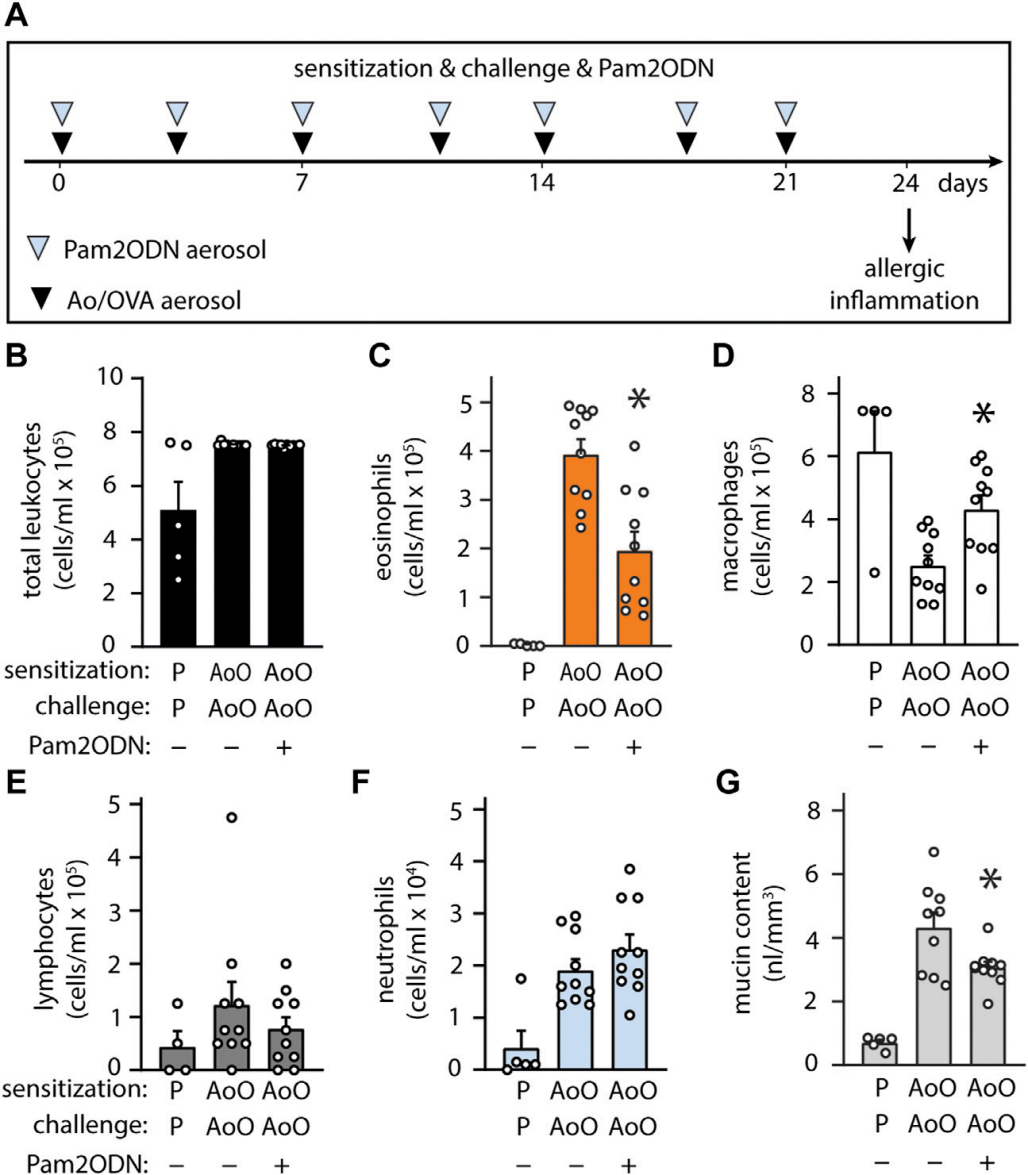
Exposure to Pam2-ODN prevents allergic inflammation to Aspergillus oryzae. **(A)** Ao-OVA experimental paradigm with a single aerosol challenge of 10 µg Ao and 2.5% OVA administered together, and repeated 7 times up for up to 21 days, at a regular interval between treatments. Pam2ODN was delivered as a separate aerosol treatment immediately before Ao-OVA with dedicated hardware. **(B–E)** Quantification of leukocytes in lung lavage for **(B)** total leukocytes **(C)** eosinophils, **(D)** lymphocytes, **(E)** macrophages, and **(F)** neutrophils (N = 4-9 mice). **(G)** Quantification of intracellular mucin content by image analysis of airway stained with PAFS (N = 4-9 mice). Bars show mean +/- SEM. p* < 0.05 by unpaired students’ *t* test for OVA vs. OP-OVA.

Allergic sensitization can occur following exposure to fungal antigens (Pashley and Wardlaw, 2021). Extending our investigations more broadly, we evaluated whether fungal allergic inflammation could also be attenuated by Pam2ODN, testing a combined Ao-OVA (AoO) model (Figure 5A), with a similar time schedule as with OVA alone (Figure 4A), except Ao-OVA or Pam2ODN were not administered from days 21–24, to avoid confounding by acute neutrophilic infiltration caused by the fungal elements or the Pam2ODN at the time of assessment. Pam2ODN treatment caused a significant reduction in inflammation (Figure 5B), predominantly due to a >50% reduction in eosinophils (Figure 5C). Macrophages were decreased in AoO-challenged mice, and this was reversed by Pam2ODN treatment (Figure 5D). Lymphocytes were not significantly elevated in AoO challenged mice, compared to mice only challenged with PBS (Figure 5E). Neutrophil levels were not increased in Pam2ODN-treated groups (Figure 5F) due to the removal of Pam2ODN treatments within 3 days of evaluation. Airway epithelial mucin content was also observed to be decreased >30% with Pam2ODN treatment (Figure 5G).

## Discussion

Over the last two decades, scientific interest in utilizing PRR agonists, particularly TLRs, for the development of novel, rapid-onset immunotherapy has grown considerably (Kirtland et al., 2020). However, these agents have not yet achieved significant clinical success, as exemplified by a recent study of a TLR9 agonist that failed to improve asthma control (Psallidas et al., 2021). The inability to translate preclinical success into clinical results is likely due to the complexity of allergic sensitization and the interplay of different variables affecting the degree of type 2 polarization. Our results suggest that the lack of clinical success with these agents may be due to the fact that the immunomodulatory efficacy of aerosolized TLR agonists is far greater when given prior to allergic stimulation rather than after. Therefore these agents may only be useful when given prophylactically at a young age to subjects at high risk for allergic asthma, rather than to subjects with established allergic asthma. In addition, prior evidence that TLR agonists can interact synergistically suggests that single agents may not offer optimal efficacy.

In an earlier study, we found that Pam2ODN had immunomodulatory effects on the chronic asthma-like disease that persists after SeV viral clearance that could not be explained by Pam2ODN-mediated antiviral properties (Goldblatt et al., 2020). In the current study, we observed Pam2ODN was broadly effective at reducing allergic inflammation and mucous metaplasia induced by three different allergens: HDM, OVA, and Ao. While each model displays unique immunogenic characteristics, SeV, HDM, OVA, and Ao all lead to a chronic lung disease driven by polarization of the lung mucosa towards type 2 immunity. The effectiveness of this treatment against all antigens evaluated, as well as the immunomodulatory effects on the SeV-induced chronic disease from our previous study, (Goldblatt et al., 2020), suggests that Pam2ODN is a broadly effective immunomodulatory agent that can regulate the type 2 immune response. It is notable that the timing of treatments and readouts varied between models, so no direct inter-model comparisons are made.

One of the most striking results is the onset speed of Pam2ODN-induced immunomodulation, with efficacy observed in as little as 24 h before HDM sensitization. Leukocyte recruitment to the lung by Pam2ODN has been measured previously to reach a peak 48 h after exposure to Pam2ODN (Alfaro et al., 2014), and thus is unlikely to be responsible for the immunomodulatory effect seen at this early time point. On the other hand, epithelial cytokine release following Pam2ODN exposure has been observed within 4 h (Alfaro et al., 2014). Additionally, it has been well established that HDM sensing by lung epithelial cells, primarily *via* TLR4, is required for the development of the full allergic phenotype (Hammad et al., 2009). We further show that lung epithelial cells can respond to HDM directly and immediately by the observation that PBS/HDM mice show accumulated intracellular mucin and no detectable eosinophil recruitment. This indicates that lung epithelial cells can generate an allergic response directly and that this can occur before the innate or adaptive leukocyte-mediated immune responses have developed. Taken together, these data suggest that integration between the type 2 immunogens and Pam2ODN immunomodulation takes place within lung epithelial cells, which are optimally positioned to be first responders to both inputs.

By varying single treatments of Pam2ODN at different time points before and after HDM sensitization, we were able to show that Pam2ODN treatment is only effective when administered before HDM sensitization. We observed Pam2ODN efficacy in as little as 24 h before sensitization, but a single treatment 2 days after sensitization was completely ineffective. These results are congruent with long-standing observations that once the initial threshold for lung anergy has been surpassed, continued exposures result in continued polarization in a type 2 direction, as a classic positive feedback loop. As the lung mucosa become progressively polarized, the window for effective therapeutic intervention diminishes.

Viewing the results from the HDM model in isolation might lead to a conclusion that Pam2ODN is simply ineffective at any time after exposure to allergen. However, when considered in conjunction with the OVA model data, a more refined picture emerges that suggests Pam2ODN immunomodulation might be specifically affecting allergic inflammation at the lung mucosa, and not systemically. Unlike HDM, ovalbumin does not readily result in allergic airway inflammation when delivered into the airways without a preceding sensitizing exposure. To achieve host sensitization, OVA is injected intraperitoneally along with a strong pro-type 2 adjuvant (i.e., alum). OVA-specific lymphocytes are processed and distributed systemically. Upon subsequent exposure, these OVA-specific lymphocytes are restimulated and provide enough pro-type-2 inflammatory stimulus to overcome the anergic threshold in the lung.

Pam2ODN is delivered directly to the airway mucosa *via* aerosol and exerts virtually no effect on systemic immune responses when evaluated in a non-allergic host (Alfaro et al., 2014). These previous data indicate that any primary action by Pam2ODN on systemic immune responses is highly implausible. Because Pam2ODN efficacy was still observed with our OVA mouse model, it seems possible that in some circumstances, immunomodulatory therapy can prevent airway polarization despite the additional type 2 immune stimulus supplied by antigen-specific lymphocytes.

Interestingly, we also observed an overall change toward less systemic immunity to OVA in Pam2ODN-treated mice, reflected by lower levels of total IgE, OVA-specific IgE and IgG2a. This suggests that airway immunomodulation during repeat exposures to allergen may attenuate systemic re-sensitization and further polarization. Over time, this might enable the host immune system to redevelop a normal anergic state without continually being re-sensitized. If so, it is possible that an extended period of treatment with Pam2ODN might prevent the progression of atopic sensitivity.

We also demonstrate that the immunogenicity of systemically sensitized leukocytes can vary significantly, and if sufficiently strong, can overcome the Pam2ODN-induced immunomodulation. We developed a novel adaptation of the acute HDM model based on intraperitoneal injection of HDM extract, followed by airway HDM challenges. Unlike our observations with the OVA model, Pam2ODN airway immunomodulation was ineffective at preventing airway allergic polarization. One possible explanation for this discord is that HDM is intrinsically a stronger type 2 immunogen than OVA, and that the HDM-specific T cells provide a stronger pro-Th2 stimulus than OVA-specific T cells. In this setting, the strength of Pam2ODN immunomodulation as a counter-regulatory stimulus is insufficient to prevent a shift to type 2 polarization. HDM immunogenicity strength is further supported by the previous observations that HDM extract is capable of sensitizing hosts directly in the lung mucosa and does not require adjuvant pairing. This hypothesis will be explicitly tested in future work.

Because different allergens appear to have different immunogenic strength, it is plausible that effective immunomodulation of strong immunogens require equivalently strong immunomodulatory stimulus. Any single PRR agonist will ultimately be restricted by receptor saturation, and thus cannot invoke a stronger response above a set threshold concentration. However, Pam2ODN provides an important clue to bypassing this immunomodulatory strength limit by pairing immunomodulatory agents that exhibit synergy. Previous work has shown that Pam2 and ODN cooperate synergistically to mediate protection against microbial pathogens, suggesting the importance of coincidence detection of multiple PRRs for innate immune sensing and generating antimicrobial responses, (Cleaver et al., 2014), and potentially highlighting a way to increase the strength of immunomodulatory responses. Our results confirm that synergistic interaction is also required for Pam2ODN efficacy against inflammation, as neither Pam2 nor ODN showed any efficacy when delivered alone. We believe this highlights the importance of evaluating combinations of immunomodulatory agents for future human studies. While Pam2 and ODN were the strongest combinations of PRR agonists for antimicrobial efficacy, (Duggan et al., 2011), a systematic screen has never been performed that assessed immunomodulatory strength, and it is possible that different combinations of PRR agonists might show even greater immunomodulatory strength. Relatedly, the dosing of Pam2 and ODN were optimized to maximal antimicrobial effects, but it is possible that even greater anti-allergic efficacy could be achieved by varying the dosing of these two components.

We propose that these data support a stronger focus on identifying the bi-directional signaling that occurs between systemic immune responses and mucosal tissues. Identifying these intercellular and intracellular pathways would enable better rational design of therapeutically relevant immunomodulatory agents. An ideal therapeutic strategy might utilize multiple agents that interact synergistically to maximally oppose any allergic stimuli that might be encountered. Additionally, we show here that allergic polarization of mucosal tissues can proceed independently of the systemic immune system. Taken together, this suggests integration of pro-allergic type-2 stimuli and the Pam2ODN immunomodulatory response. Thus, it is plausible that treatments focused on reducing type 2 polarization of the mucosal tissue might be as effective as broadly blocking systemic immune responses (e.g., glucocorticoids), while likely avoiding toxicities of systemic immunosuppression.

The current study has important limitations that impact its interpretation. Most notably, the experimental models of allergic inflammation studied (HDM, OVA, Ao) were executed with differing times between Pam2ODN treatment and the allergic stimulus. Similarly, the time from allergic stimulus to certain readouts were not uniform across the models. Finally, some readouts (e.g., allergen-specific IgE) were available for some models but not others. The principle consequence of these differences is rendering head-to-head comparisons of the inter-model effects of Pam2ODN inappropriate. The presented data demonstrate desirable effects of Pam2ODN in all three tested models (as well as in previously in SeV-induced allergic inflammation [REFS]), but the current work does not establish that the mechanisms are the same between the models. Indeed, the efficacy of Pam2ODN in preventing or attenuating allergic inflammation does appear to vary temporally between models. These differences are an active area of investigation. In conclusion, we have shown that Pam2ODN is a generalizable immunomodulatory agent that counteracts allergic type 2 immune responses caused by a wide variety of immunogens and is likely superior to previously tested single agents, due to synergistic cooperation. The public need for immunomodulatory agents that can counteract the morbidity of type 2 allergic disease is great, and clinical studies with Pam2ODN have already shown the drug to be well tolerated in humans (NCT04313023, NCT04312997, NCT03794557, NCT02566252, NCT02124278), allowing Pam2ODN to be efficiently evaluated in the setting of allergic immunotherapy.

## Materials and methods

### Mice

Animal studies are reported in compliance with the ARRIVE guidelines (Kilkenny et al., 2010). Breeder BALB/cJ mice were obtained from the Jackson Laboratory (Sacramento, CA) and housed in specific pathogen-free conditions on a 12-hour light/dark cycle with free access to food and water. For euthanasia, mice were injected intraperitoneally with 2,2,2-tribromoethanol (250 mg/kg) and exsanguinated by transection of the abdominal aorta. For all mouse experiments, all subjects in a cohort were of the same sex. Each experiment was repeated a minimum of 3 times. For each experiment, at least one replicate performed with each sex. We did not observe any significant differences between sex in our study, though this approach was not powered to exclude the possibility of very subtle differences. All procedures were performed in accordance with the Institutional Animal Care and Use Committee of MD Anderson Cancer Center and the Texas A&M Institute for Biosciences and Technology. Chemicals were obtained from Sigma-Aldrich (St. Louis, MO) unless otherwise specified.

### Treatment with aerosolized Pam2ODN

This was performed as described (Alfaro et al., 2014). Briefly, ODN 5′ TCG TCG TCG TTC GAA CGA CGT TGA T 3’ as the sodium salt on a phosphorothioate backbone (ODN M362) was purchased from TriLink BioTechnologies (San Diego, CA) and 2,3-bis (palmitoyloxy)-2-propyl-Cys-Ser-Lys-Lys-Lys-Lys-OH (Pam2CSK4) as the trifluoroacetic acid salt was purchased from Peptides International (Louisville, KY). A solution of ODN (1 μM) and Pam2CSK4 (4 μM) in endotoxin-free sterile water (8 ml) was placed in an Aerotech II nebulizer (Biodex Medical Systems, Shirley, NY) driven by 10 L/min of 5% CO_2_ in air to promote deep breathing. The nebulizer was connected by polyethylene tubing (30 cm × 22 mm) to a 10-L polyethylene chamber vented to a biosafety hood. Mice were exposed to the aerosol for 20 min, resulting in the nebulization of ∼4 ml of O/P solution. The optimal molar ratio between Pam2 and ODN was previously determined (Evans et al., 2011). The dosage of Pam2ODN was chosen to reflect the upper shoulder of secreted epithelial cytokines, leukocyte recruitment, and anti-microbial efficacy by dose-response experiments (Alfaro et al., 2014). The dosage, route, and overall process of administering a Pam2ODN treatment was standardized across every experiment in the study. The only variation to Pam2ODN treatment occurs in the studies measuring additivity and synergy of the independent ligands, whereby some groups received only ODN or only Pam2 components in the nebulized solution, but other wise were administered in exactly the same way and at the same final concentrations as the combined solution.

### Sensitization and airway challenge with HDM

Lyophilized HDM extract (Stallergenes Greer, Lenoir, NC) was resuspended in PBS. Mice were sensitized to 100 μg of HDM (protein weight) by depositing 40 μL of reconstituted HDM into the oropharynx and allowing aspiration into the lungs, with mice suspended by the upper incisors on a board at 60° from horizontal under isoflurane anesthesia. Mice were then challenged with 10 µg of HDM by depositing the same volume of reconstituted HDM into the nasal vestibule. This model and HDM dosages were selected from previously published data and confirmed by us during pilot studies (Supplementary Figure S1). In one case, mice were sensitized to 100 µg of HDM by intraperitoneal injection of 40 ul of reconstituted HDM. For all experiments, mouse age at the time of sensitization to HDM was between 6 and 7 weeks old.

### Sensitization and airway challenge with OVA

Mice were sensitized to 20 g ovalbumin (OVA) (Grade V, 2.25 mg alum in saline, pH 7.4; Sigma, St. Louis, MO) by intraperitoneal (i.p.) injection on day 0 and 7. Sensitized mice were exposed for 30 min to an aerosol of 2.5% (wt/vol) ovalbumin in PBS, using an Aerotech II nebulizer, as described above.

### Airway challenge with Aspergillus oryzae and OVA

Aspergillus oryzae protease (Sigma-Aldrich, St. Louis, MO) was resuspended in PBS. Mice were challenged with 10 ug A. oryzae and 2.5% (wt/vol) OVA by aerosol 2.5 times per week for 3 weeks. A. oryzae dose was selected from previously published data (Morgan Knight et al., 2018). Aerosol challenge was performed as described above for OVA.

### Bronchoalveolar lavage and differential leukocyte analysis

This was performed by instilling and collecting two 1 ml aliquots of ice-cold PBS through a 20 gauge cannula inserted through rings of the exposed trachea of euthanized animals, then combining the aliquots as described (Alfaro et al., 2014). Total leukocyte count was determined using a hemocytometer, and differential counts by cytocentrifugation of 100 μL of lavage fluid at 300 g for 5 min followed by Wright-Giemsa staining.

### Histochemistry

Lungs were fixed by intratracheal inflation with 10% formalin to 20 cm H_2_O pressure for 12 h, and then embedded in paraffin. Tissue blocks were cut into 5-µm sections, mounted on frosted glass slides (Medline, Northfield, IL), deparaffinized with xylene, washed with ethanol, then rehydrated and stained with hemoxtylin and eosin (H&E) (Sigma-Aldrich, St. Louis, MO).

### Epithelial mucin content

Epithelial mucin content was measured as described (Evans et al., 2004; Tuvim et al., 2009b; Piccotti et al., 2012). Lungs were fixed and processed, as described above, but stained with periodic acid fluorescent Schiff reagent (PAFS). Left-axial bronchus sections were obtained using a custom precision cutting instrument (ASI Instruments, Warren, MI), as previously described (Goldblatt et al., 2020). Images were acquired by investigators blinded to mouse treatment, and morphometric analysis of the images for quantitation of intracellular mucin was performed using MATLAB Software (Mathworks Software, Natick, MD). Data are presented as the area of intracellular mucus, normalized to the length of the basement membrane.

### Statistical analysis

All data sets were first analyzed with the Shapiro-Wilk test to determine normality. For analyses where there was only a single comparison between two groups, data was analyzed by Student’s t-test or Mann-Whitney *U* test for normally and non-normally distributed data, respectively. For analyses where multiple experimental groups were compared against a single control, data were first analyzed by one-way ANOVA or ANOVA on ranks to determine if a significant difference between any groups was present for normally and non-normally distributed data, respectively. If a significant difference was found by ANOVA, data were further analyzed using Dunnett’s test. For analyses where each experimental group was compared to every other group, data were further analyzed using Tukey’s test for one-way ANOVA or Dunn’s test for all pairwise combinations for ANOVA on ranks. Significance was determined from adjusted *p* values. For studies comparing Pam2 and ODN individually and together, an interaction score from linear regression analysis was used to determine whether there was a synergistic effect. All data were analyzed using Prism (version 9, GraphPad Software, San Diego, CA). *p* < 0.05 was considered statistically significant.

## Data Availability

The raw data supporting the conclusion of this article will be made available by the authors, without undue reservation.
