# Supplementary material for: Epithelial immunomodulation by aerosolized Toll-like receptor agonists prevents allergic inflammation in airway mucosa in mice
Source: Front Pharmacol. 2022 Aug 29;13:833380. doi: 10.3389/fphar.2022.833380 (PMC9464972; doi:10.3389/fphar.2022.833380)
Supplement: Supplementary file 1 [file DataSheet1.docx]

Supplementary Material

# Supplementary Figures and Tables

#
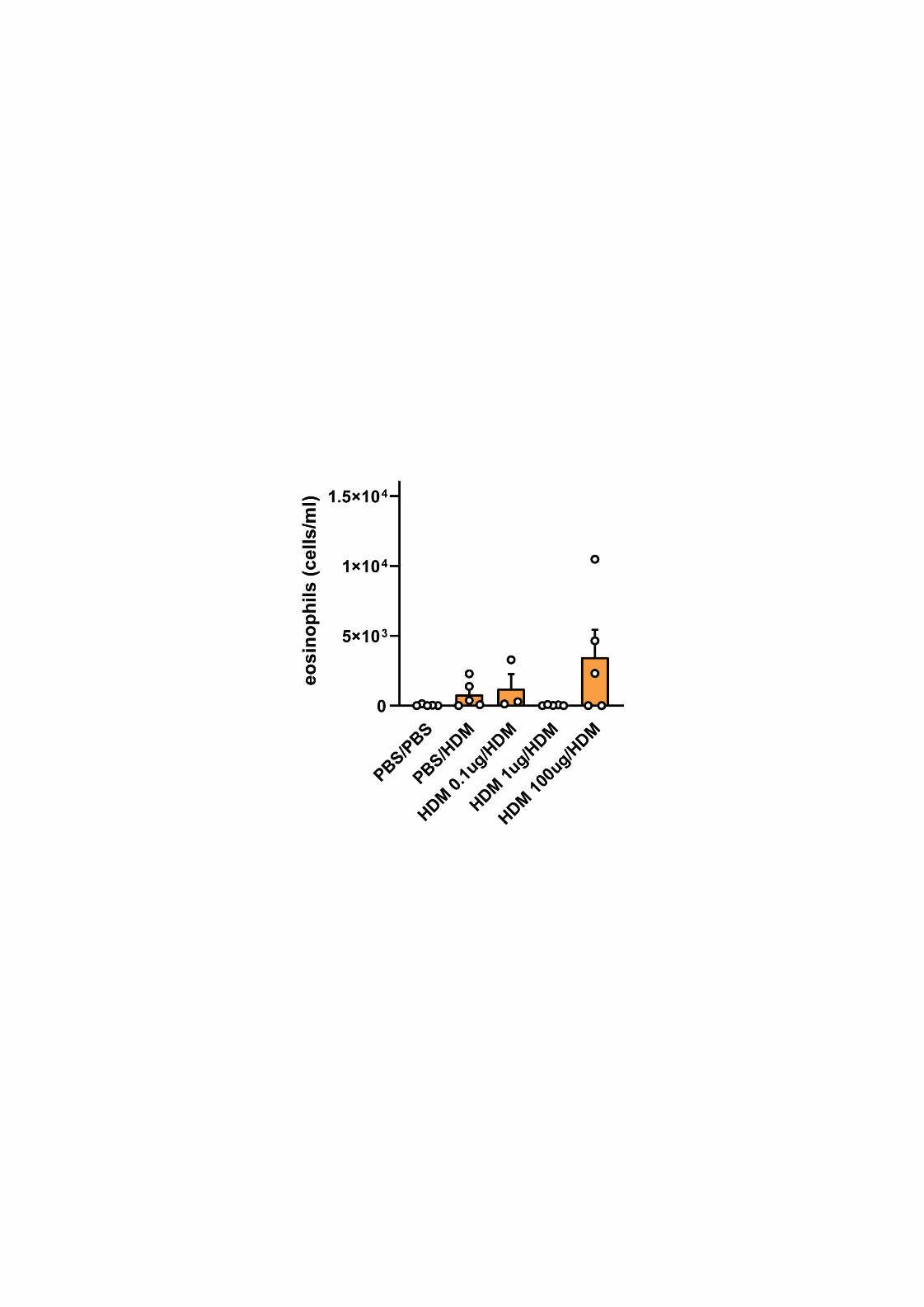


# Supplementary Figure 1. **HDM pilot studies.** Mice were sensitized to HDM at doses indicated, and then challenged with 10 ug HDM from day 7 to day 12. Lung inflammation was assessed at day 15.


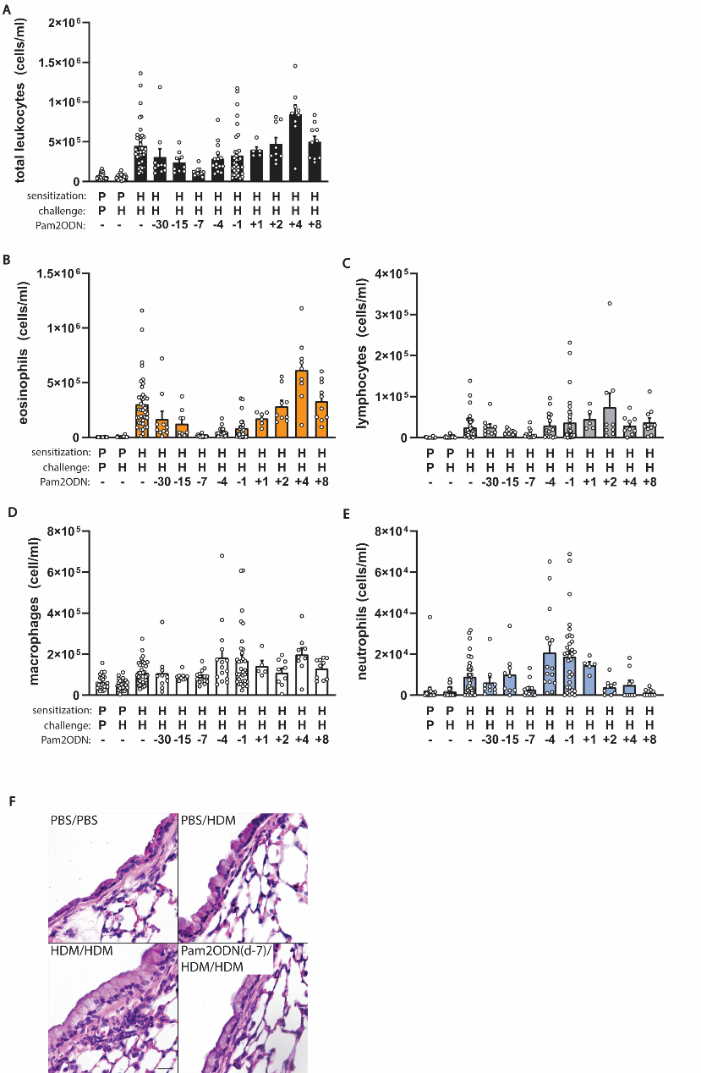


**Supplementary Figure 2.** Pam2ODN attenuates allergic inflammation to HDM. In these studies, Pam2ODN treatment was delivered either as a single exposure after HDM sensitization or as a series of 6 daily treatments either immediately after initial HDM sensitization (days 1 through 6) or concurrently with HDM challenge (days 7:12). (A) Total leukocytes, (B) eosinophils, (C) lymphocytes, (D) macrophages, and (E) neutrophils trends obtained from concatenating data from multiple experiments. Statistics were not applied to this data because they do not represent individual experiments. (F) Pam2ODN treatment was administered 7 days before sensitization of HDM and lungs stained for H&E to inflammation and tissue morphology. Scale bar = 20 µm.


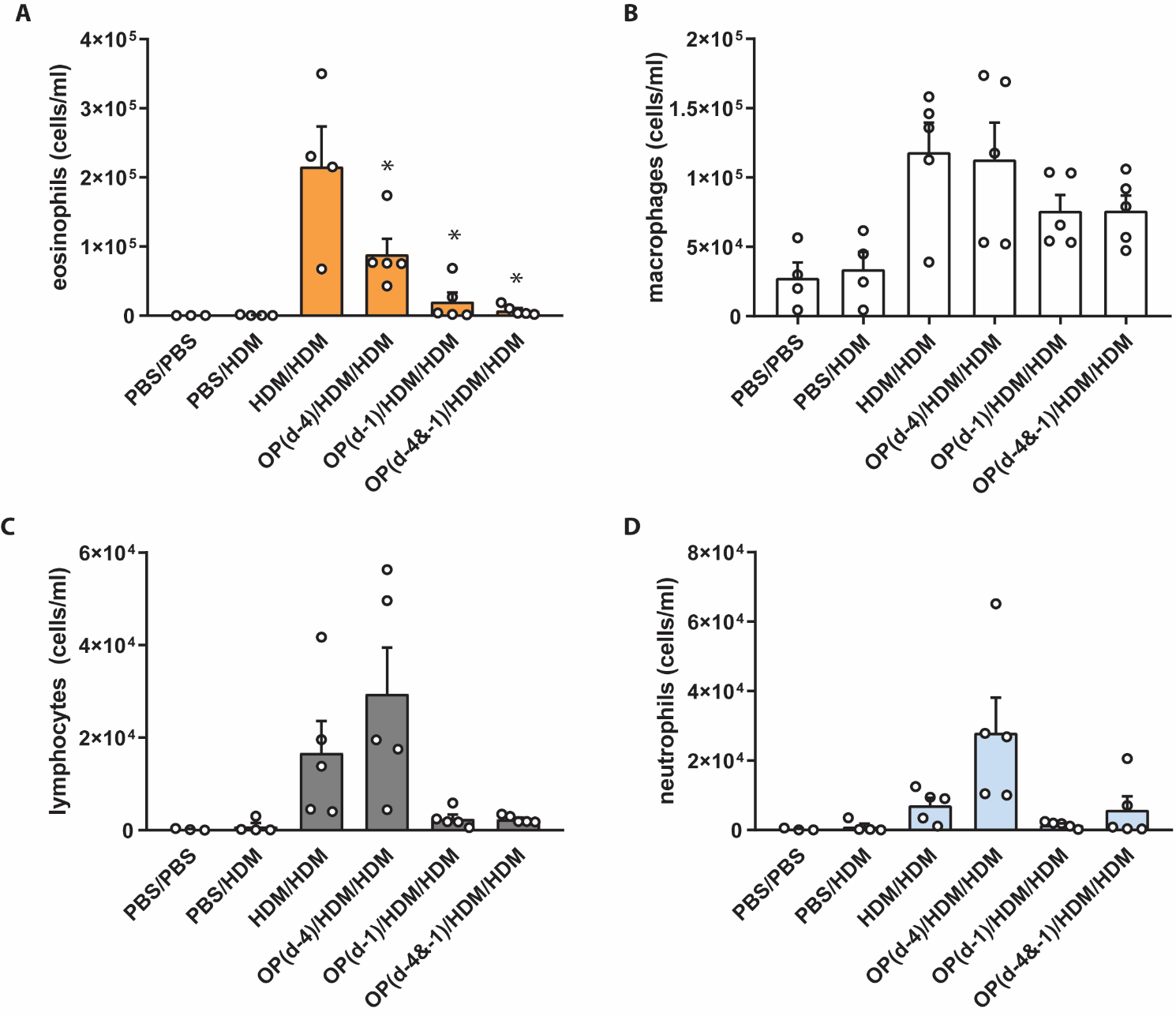


Supplementary Figure 3. Multiple Pam2ODN treatments are better than a single treatment. Pam2ODN treatment 4 days and 1 days before HDM sensitization was superior to Pam2ODN treatment on either day alone.


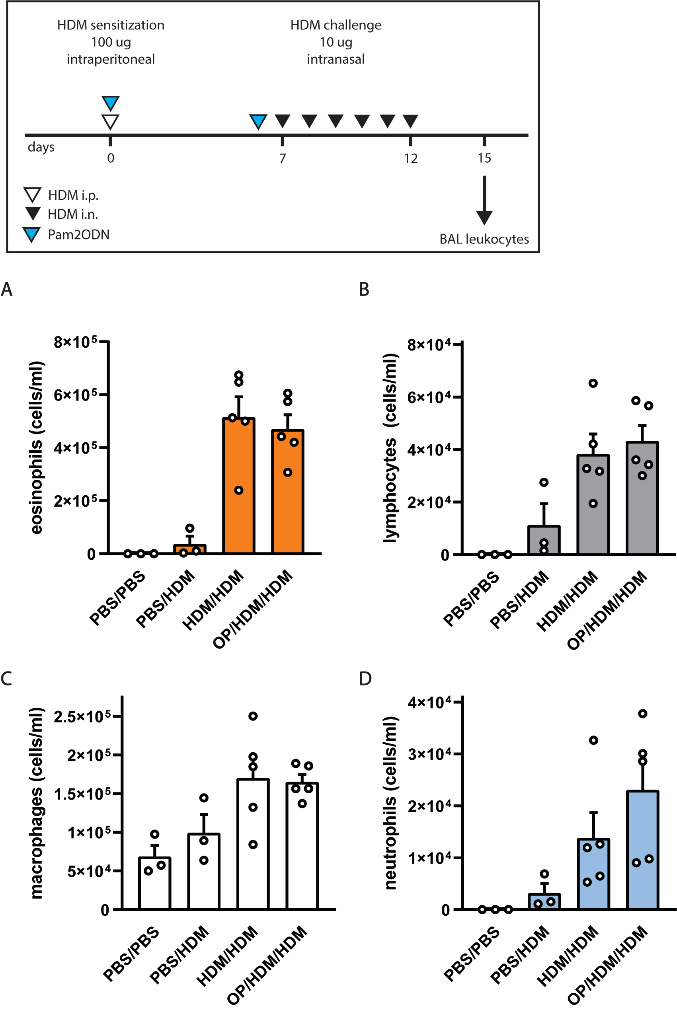


Supplementary Figure 4. **Pam2ODN does not attenuate HDM if systemically sensitized.** Pam2ODN treatment on day 0 and day 7 does not prevent allergic inflammation to HDM when host is sensitized to 100 μg HDM intraperitoneally.
